# Translating research into action: The design and development of an Indigenous specific suicide intervention skills training program (I‐ASIST)

**DOI:** 10.1111/ajr.12903

**Published:** 2022-07-14

**Authors:** Bushra Farah Nasir, Stephen Kisely, Leanne Hides, Sharon Brennan‐Olsen, Srinivas Kondalsamy‐Chennakesavan, Geoffrey C. Nicholson, Neeraj S. Gill, Gavin Beccaria, Maree Toombs

**Affiliations:** ^1^ Toowoomba Regional Clinical Unit, Medical School, Faculty of Medicine The University of Queensland Toowoomba Qld Australia; ^2^ Princess Alexandra Hospital Southside Clinical Unit, Faculty of Medicine The University of Queensland Brisbane Qld Australia; ^3^ School of Psychology, Faculty of Health and Behavioural Sciences The University of Queensland Brisbane Qld Australia; ^4^ School of Health and Social Development Deakin University, Geelong Waterfront Campus Geelong Vic. Australia; ^5^ Institute for Health Transformation Deakin University, Geelong Waterfront Campus Geelong Vic. Australia; ^6^ Faculty of Health University of Canberra Canberra ACT Australia; ^7^ Toowoomba Regional Clinical Unit, Faculty of Medicine The University of Queensland Toowooomba Qld Australia; ^8^ School of Psychology and Counselling University of Southern Queensland Toowoomba Qld Australia; ^9^ Faculty of Medicine The University of Queensland Herston Qld Australia

**Keywords:** Aboriginal and Torres Strait islander, Indigenous Australians, suicide, suicide intervention, suicide prevention

## Abstract

Note: *We respectfully refer to Aboriginal and Torres Strait Islander people as Indigenous in this study*.

**Objective:**

To design and develop an Indigenous specific suicide intervention skills program that focuses on education and intervention training as an effective suicide prevention strategy.

**Method:**

Using a co‐designed wrap‐around framework, we developed a program in collaboration with >90 communities, stakeholders and service providers across Australia to understand knowledge, awareness and sense of connectedness between at‐risk groups and health services or support groups.

**Results:**

The I‐ASIST training provides participants with the necessary skills and knowledge to apply a suicide intervention model. The framework behind the intervention model provides caregivers the awareness to recognise when someone may be at risk of suicide. It then gives them the skills to connect with a person at risk of suicide and to understand and clarify that risk, steps to keep that person safe for a specific period and then provide them with the resources or links required for further help. The program enables the development of knowledge through interactive strategies through cultural recognition and empowerment of participants. Based on a social‐enterprise model, I‐ASIST has been translated into a certified program supported by LivingWorks Australia.

**Conclusion:**

Based on a strengths‐based and self‐determination model of co‐design, this grass roots innovative framework creates suicide safer communities.

**What is already known in this subject:**

- Suicide is the leading cause of death for Indigenous children aged 5–17 yo, and those aged 15 to 44 experience twice the rate of their non‐Indigenous counterparts
- Despite high prevalence, there are no specifically tailored Indigenous specific suicide intervention training programs

**What this study adds:**

- This study describes the development of an Indigenous specific suicide intervention skills training program co‐designed in consultation with Indigenous communities
- It highlights the foundational program, leading to a nation‐wide collaboration with LivingWorks Australia

## 1 INTRODUCTION

Suicide is the second leading cause of death for Indigenous children aged 14 years and less – they are up to eight times more likely to die by suicide than their non‐Indigenous peers.^1^ Furthermore, suicide is the leading cause of death for both 5‐ to 17‐year‐old Indigenous youth^1^ and Indigenous youth aged 15–25 years.^2^ The suicide rate in Indigenous people aged 25–44 years is twice that of non‐Indigenous Australians.^1^ Additionally, for every suicide, there are many more attempted suicides and incidences of self‐harm.^3^ Contributing factors include social marginalisation, racism, disempowerment, historical intergenerational trauma and experiences that influence high rates of trauma and mental illness.^4^, ^5^

Despite these concerning statistics, there are limited examples of culturally appropriate suicide prevention programs for Indigenous communities in Australia,^6^, ^7^although a systematic review did find that gatekeeper training is a promising intervention.^7^ Gatekeeper training has some integral strengths, as it can be tailored to different communities by addressing issues specific to the setting. This training upskills community members with existing personal connections to learn how to identify warning signs and keep people at‐risk safe in the short term. Evidence for the effectiveness of gatekeeper training has identified short‐term positive gains in knowledge, attitudes, self‐efficacy and perceived skills in the general population.^8^ Large‐scale community‐based, multimodal suicide prevention strategies with gatekeeper training as a core component have shown significant reductions in suicide mortality and suicide attempts among young people in the 2 years after implementation, compared to those in control conditions.^9^, ^10^ However, programs evaluating the impact of gatekeeper training within Indigenous communities are scarce.^7^ Furthermore, programs that are co‐designed and led by Indigenous people themselves providing necessary empowerment and ownership^11^ are currently absent.

This study describes the design, development and evaluation of an Indigenous specific suicide intervention skills program, and how key learnings from this study were used to translate the model into a world‐first, strength‐based, certified suicide intervention training program specifically for Indigenous people.

## 2 METHODOLOGY

A strategic approach towards Indigenous suicide prevention was implemented nationally in Australia in 2013.^12^ The Indigenous Network Suicide Intervention Skills Training (INSIST) Program was co‐designed and developed through an in‐depth and widespread consultation process conducted over 2 years.

## 3 THE INSIST PROGRAM

The Indigenous Network Suicide Intervention Skills Training (INSIST) program is a multi‐faceted gatekeeper (‘*responder’*) suicide intervention training program, designed in consultation with Indigenous communities, delivered by Indigenous trainers for Indigenous communities across Australia. The program was co‐designed and developed through an in‐depth and widespread consultation process conducted over 2 years. See Nasir et al (2017)^6^ for a full description of this process. This community‐led frontline suicide prevention strategy focused on the social, emotional, cultural and spiritual wellbeing of Indigenous people. Using a strength‐based approach, the training provides individuals the skills to recognise when someone is thinking of suicide, take appropriate action to connect and understand the risk, keeping people immediately safe and helping them access further help. The focus of INSIST was to implement a bottom‐up approach to training, to maximise community involvement in the training, to ensure its long‐term effectiveness, efficacy and uptake. The program framework is intuitive and designed in a way that enables caregivers to respond to suicide risk effectively.

In 2017, the INSIST program was launched and commenced delivery across rural, regional and remote locations of South‐East and Central Queensland. Originally funded to train 100 responders, the training program expanded its reach using a social enterprise model.^13^ The social enterprise model enables benefits for trainers by providing social purpose, maximising social impact alongside providing a source of income for trainers. As a result, 321 responders were trained across 22 workshops to keep 13 communities safe. Twenty‐four Indigenous trainers were also trained under a train‐the‐trainer model to become accredited trainers. INSIST sat under a social enterprise model having the advantage of providing necessary social benefits and ownership by Indigenous peoples. Maintaining community ownership remains a priority for those who have completed training.

### 3.1 The I‐ASIST program

**FIGURE 1:**
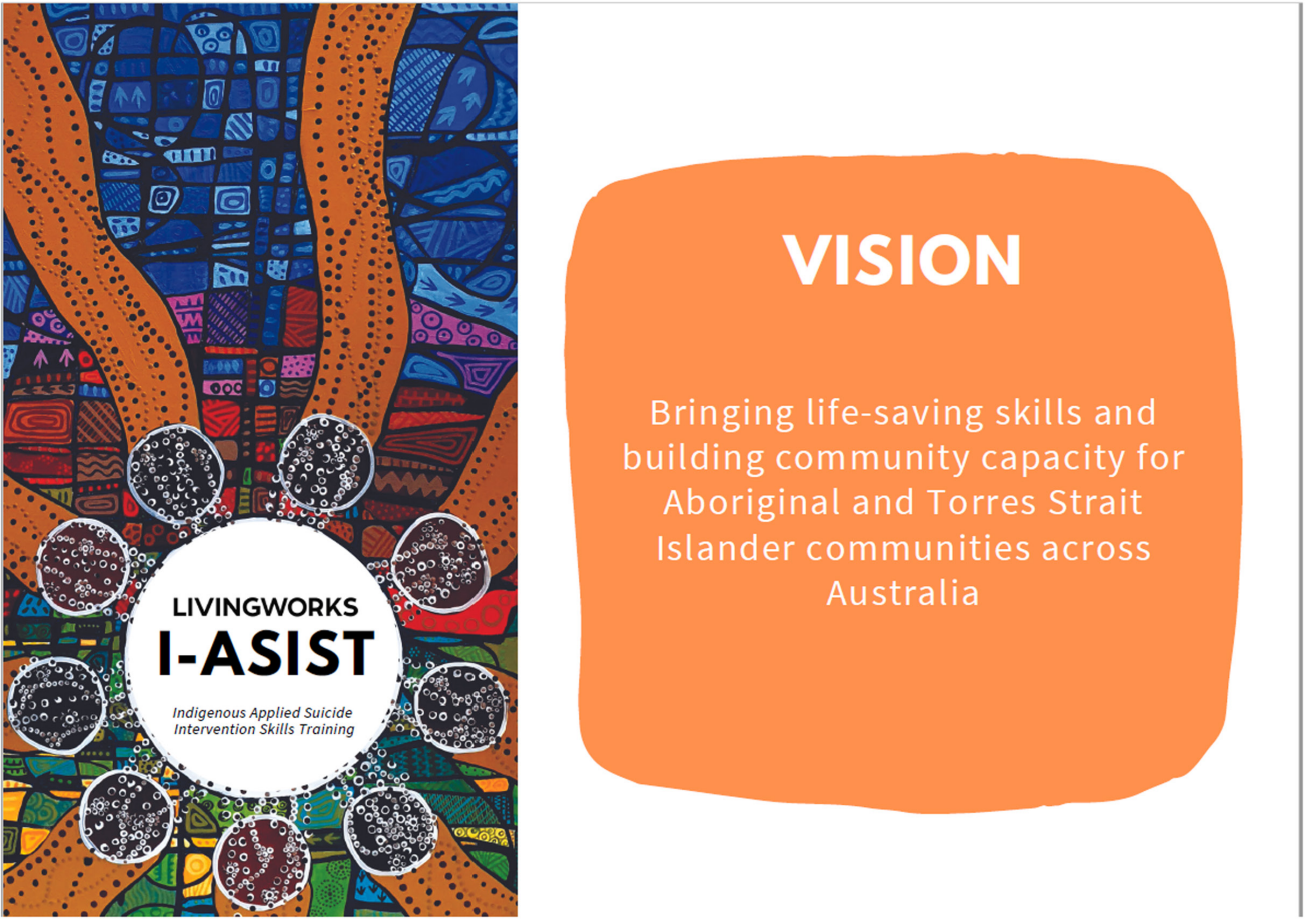
The I‐ASIST training program vision

The INSIST program formed the basis of a wider Indigenous‐Applied Suicide Intervention Skills Training program (I‐ASIST) program (Figure 1). The acronym I‐ASIST was preferred over the term INSIST by the community due to coercive undertones of the word ‘insist’ and therefore all further references to the program became I‐ASIST. I‐ASIST is now a stand‐alone, certified suicide intervention model that is provided in partnership with LivingWorks Australia.

### 3.2 Core features

**FIGURE 2:**
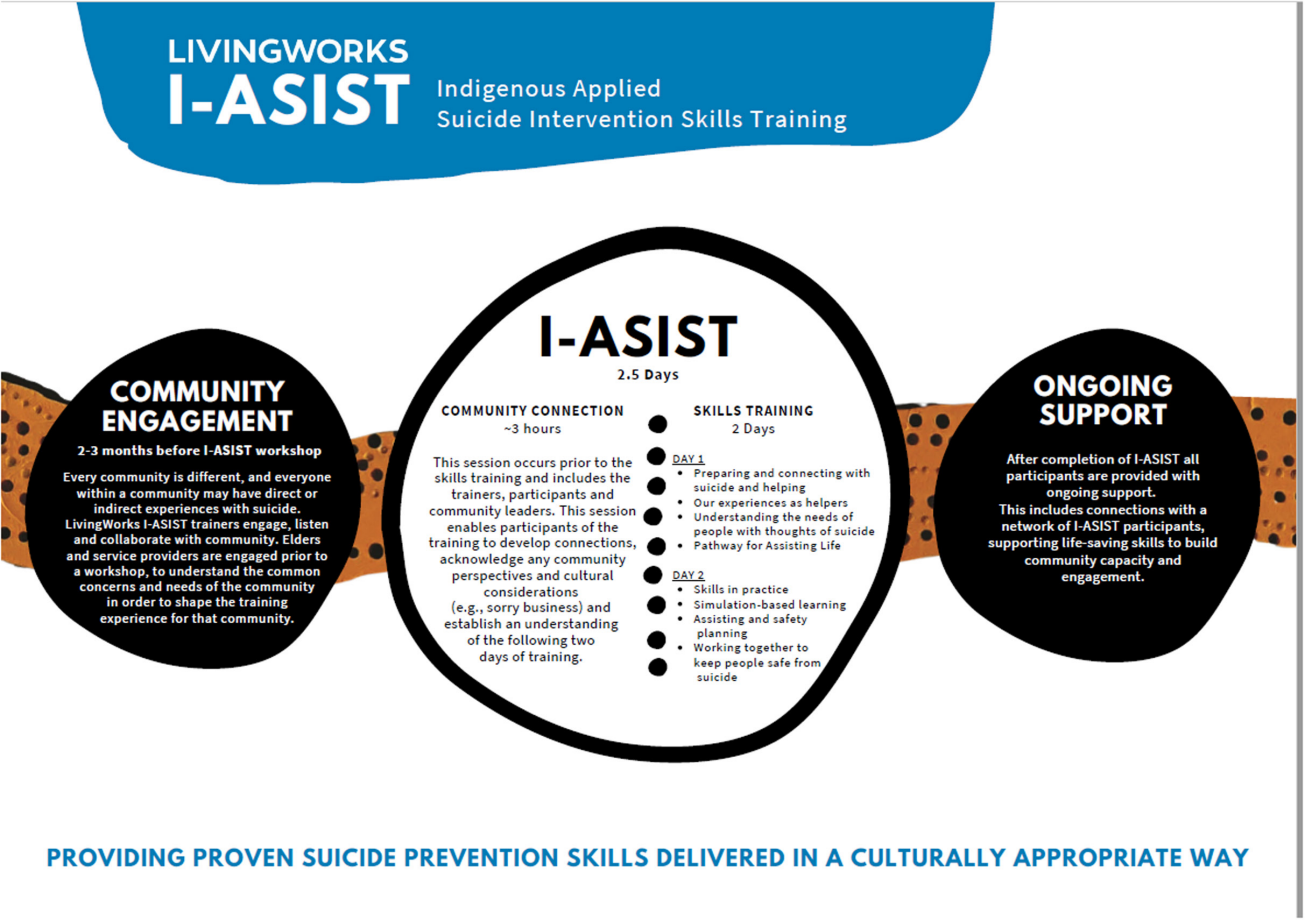
The I‐ASIST training workshop framework

I‐ASIST workshops are delivered by qualified, culturally trained Indigenous and non‐Indigenous trainers. However, the lead trainer must always be Indigenous. This is to ensure cultural safety for participants and to honour the core of the training, which is Indigenous developed and led. I‐ASIST is an interactive training program (Figure 2), which builds suicide intervention skills of responders to mitigate the immediate risk of suicide. The program is based on a scientifically proven intervention model developed by Living Works, using the Applied Suicide Intervention Skills Training (ASIST). The training can be learned and used by anyone – there are no prior skills required. Anyone 16 years or older, with basic literacy skills can become an I‐ASIST responder. Because the training is comprehensive and does not rely on prior qualifications or knowledge, responders can become professional responders. The training has been designed specifically for Indigenous responders but as mentioned previously is also appropriate for non‐Indigenous responders who have worked and are accepted in Indigenous communities if they are the secondary trainer to the Indigenous trainer. The program recognises the importance of social and emotional well‐being concepts and embeds these core principles of practice within its training.^14^

A 3‐h pre‐workshop ‘Community Care Day’ is included to allow participating responders to understand the purpose of I‐ASIST. It is an informal ‘yarning’ session where responders get to know each other and understand what the next 2 days of the training will entail. Talking about suicide may bring up confronting feelings, memories or emotions,^14^ and participants can opt‐out of the training if they feel they are not ready on that day. They are welcome to return to a later training session. Participants are informed of their role in the community once they have this training, which is to create suicide safer communities.

The 2‐day I‐ASIST workshop provides necessary skills and a comprehensive overview of suicide first aid. It is a requirement that at least one trainer at any given workshop is an Indigenous trainer. This Indigenous trainer is most often the lead trainer running the workshop. To become registered, trainers must complete an intensive course called an I‐ASIST Training for Trainers (T4T), present workshops regularly and submit continuous quality assurance reports. All trainers receive ongoing support from LivingWorks and the I‐ASIST Network Advisory Group, as they work to build suicide‐safer Indigenous communities.

The I‐ASIST program aims to evaluate the connectedness and information flow between trained participants. Social Network Analysis (SNA) data were collected and will be used to examine and quantify connectedness and information flow/diffusion between at‐risk people, gatekeepers, community and external support agencies. SNA is based on the theory that relationships influence behaviour, and social networks in particular play a key role in influencing behaviour.^15^ SNA has been used previously in suicide prevention research^16^ and provides useful information in the changes of relationships, while visually demonstrating and quantifying connectedness to show the effectiveness of the training program and referral patterns after the training.

## 4 CONCLUSION

The I‐ASIST Program is a multi‐faceted suicide intervention training program that aims to increase knowledge and awareness of suicide risk factors by developing a sense of connectedness between at‐risk groups and health services or support groups. Participants trained to become ‘responders’ are upskilled to improve their willingness and skill to intervene and refer at‐risk people to appropriate support and service. This program aimed to address three national health priority areas: Indigenous health, mental health and suicide prevention. In particular, and for the first time, this study was designed to consolidate and maintain changes in gatekeeper knowledge and confidence.

The co‐design approach of the program has led to successful uptake, and provided strong evidence on value, sustainability and behaviour. The program focuses on cultural importance and empowerment for Indigenous communities. The program has enriched people, built community capacity and provided a long‐term sustainable model of suicide prevention. Future evaluation of the program will aim to provide an understanding on the connectedness developed because of changes in knowledge, awareness and skills of responders who have completed the I‐ASIST program.

## AUTHOR CONTRIBUTIONS

BN: conceptualization; data curation; investigation; methodology; project administration; visualization; writing – original draft; writing – review and editing. SK: conceptualization; funding acquisition; investigation; methodology; writing – review and editing. LH: conceptualization; funding acquisition; investigation; methodology; writing – review and editing. SB‐O: conceptualization; funding acquisition; investigation; methodology; writing – review and editing. SK: conceptualization; funding acquisition; investigation; methodology; project administration; writing – review and editing. GN: conceptualization; funding acquisition; investigation; methodology; project administration; writing – review and editing. NG: conceptualization; funding acquisition; investigation; methodology; writing – review and editing. GB: conceptualization; funding acquisition; investigation; methodology; project administration; writing – review and editing. MT: conceptualization; funding acquisition; investigation; methodology; project administration; visualization; writing – original draft; writing – review and editing.

## FUNDING INFORMATION

This research was conducted as part of the Indigenous Network Suicide Intervention Skills Training (INSIST) program run by The University of Queensland Rural Clinical School, Faculty of Medicine with funding from the National Health and Medical Research Council (NHMRC) Australia (APP1076729).

## ETHICS APPROVAL

The University of Queensland Human Research Ethics Committee approved this study (Approval #: 2015000662).

## CONFLICT OF INTEREST

The authors declare no conflicts of interest.
